# Left atrial wall imaging using a novel black-blood late gadolinium enhancement sequence

**DOI:** 10.1186/1532-429X-18-S1-P62

**Published:** 2016-01-27

**Authors:** Maxine Tang, Tamer Basha, Sophie Berg, Kraig V Kissinger, Beth Goddu, Warren J Manning, Reza Nezafat

**Affiliations:** 1 grid.239395.70000000090118547Cardiology, Beth Israel Deaconess Medical Center, Boston, MA USA; 2 grid.239395.70000000090118547Radiology, Beth Israel Deaconess Medical Center, Boston, MA USA

## Background

**Figure 1:**
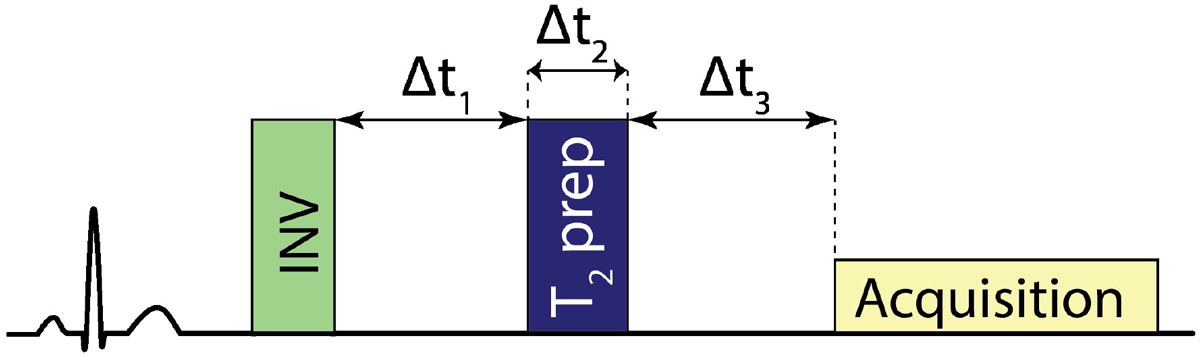
**Schematic of the black-blood LGE sequence**.

Late gadolinium enhancement (LGE) is commonly used clinically to identify myocardial infarct or scar as a hyper-enhanced region. However, the blood pool also remains bright in such images, masking adjacent bright tissue. LGE has also been used extensively to image fibrosis in the left atrium (LA) in patients with atrial fibrillation. However, identification of LA wall in LGE is challenging due to the thin wall of LA and high signal of the blood pool in the LA. We recently developed a novel black-blood LGE (BB-LGE) sequence (Fig. 1) which uses an optimized combination of inversion-recovery and T_2_-preparation (T_2_-prep) prepulses to simultaneously null the healthy myocardium and blood signals by leveraging the higher T_2_ of blood. In this study, we sought to assess the feasibility of BB-LGE in imaging of LA.

## Methods

Seven patients (6 male, 65+/-17 years) with a history of atrial fibrillation were recruited to participate in an IRB-approved study to compare BB-LGE vs. LGE for imaging of the atrial wall. All images were acquired at 1.5T (Philips Achieva). LGE and BB-LGE scans were acquired 15-20 minutes after injection of 0.1 mmol/kg of MultiHance contrast agent. Both sequences were acquired with identical imaging parameters and spatial resolution; BB-LGE required an additional T_2_-prep to null the blood signal. The parameters of the BB-LGE sequence (Δt1, Δt2, Δt3 in Fig. 1) were determined in a short ~30 sec navigator scan by a technologist prior to the BB-LGE scan. Images were subjectively scored by an observer according to percentage of the LA border that was clearly defined. A score of 1 was given for scans in which greater than 75% of the border was well-defined. A score of 2 was given for 50-75%, a score of 3 was given for 25-50%, and a score of 4 was given for less than 25% of the LA border. The scores for each sequence were compared using a paired T-test analysis.

## Results

**Figure 2:**
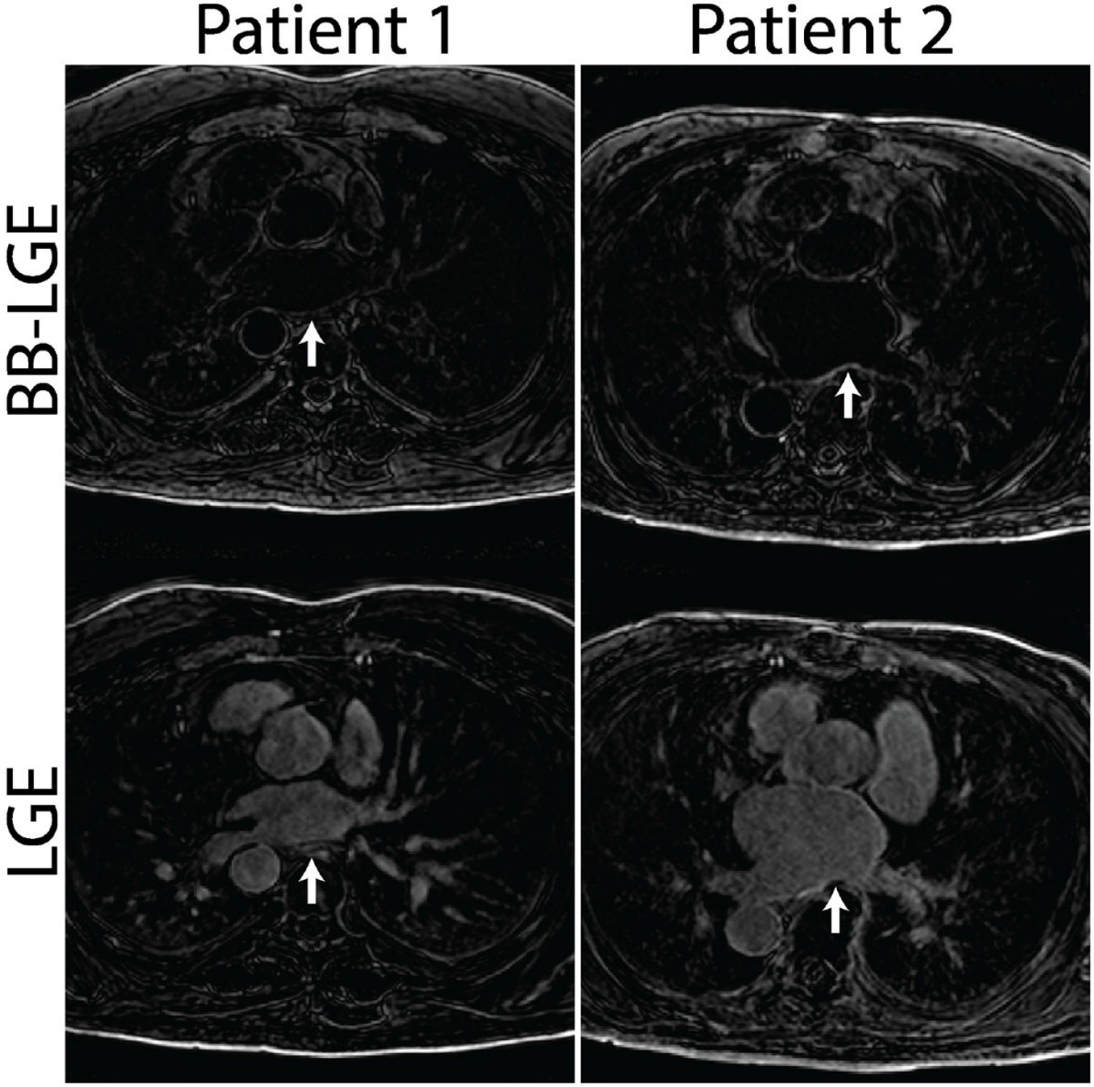
**Side-by-side comparison of similar slices showing the left atrium (indicated by white arrow) in three separate patients imaged with both the BB-LGE and LGE sequences**.

Figure 2 shows examples of BB-LGE and LGE images of the LA. The LA wall could be easily delineated in BB-LGE images. There was significant difference in the scores of LA border definition for the BB-LGE images vs. the LGE images (p = 0.02). The BB-LGE image scores were lower than the LGE image scores (1.38+/-0.74 vs. 2.25+/-0.89), indicating that a higher percentage of the LA border was well-defined on the BB-LGE images.

## Conclusions

Our proposed BB-LGE sequence has the potential to provide improved visualization of the LA wall compared to LGE protocols due to its ability to simultaneously null both the healthy myocardium and blood pool signals. This preliminary data demonstrates the need for further study of this sequence in patients with suspected LA fibrosis.

